# Study on the Influence of Metal Ions on the Dispersion of Fine Calcium Gangue Minerals

**DOI:** 10.3390/molecules27248963

**Published:** 2022-12-16

**Authors:** Zhongyi Liu, Jie Liu, Yinfei Liao, Zilong Ma, Chenxi Jin

**Affiliations:** 1 National Engineering Research Centre of Coal Preparation and Purification, China University of Mining and Technology, Xuzhou 221116, China; 2 Sinopec Ningbo Engineering Co., Ltd., China University of Mining and Technology, Ningbo 315103, China; 3 College of Environmental Science and Engineering, Tongji University, Shanghai 200092, China

**Keywords:** metal ions, fine, calcite, dispersion behavior

## Abstract

In this study, the calcium gangue material calcite (−10 μm) was used to investigate the effects of different kinds of metal ions and dosages on the dispersion behavior of calcite. The test results showed that the dispersion behavior of calcite was poor under strongly alkaline conditions without the addition of metal ions, and the reason for that was calcite dissolved ions. The degree of influence of different metal ions on calcite dispersion behavior was Fe^3+^ > Mg^2+^ > Na^+^. The three metal ion dosage tests showed that the dispersion behavior of calcite became poorer with the increase of metal ion dosage. This mainly showed that with the increase of Na^+^ dosage, the trend of the dispersion behavior of calcite was not obvious, but with the increase of Fe^3+^ and Mg^2+^ dosage, the trend of calcite dispersion behavior changed more. The dispersion behavior of calcite was devastated by 5 × 10^−4^ mol/L Fe^3+^ at pH = 4–12. The different mechanisms of the three metal ions were identified by zeta potential, solution chemistry, and XPS analysis. Na^+^ only changed the zeta potential value of the calcite surface, which acted as a compressed electric double layer. However, the formation of metal hydroxide species or metal hydroxide surface precipitation due to the adsorption of Fe^3+^ and Mg^2+^ on the mineral surface resulted in the change of the dispersion behavior of calcite.

## 1. Introduction

Calcite is associated with a variety of valuable minerals [1,2], such as smithsonite [3], fluorite [4], scheelite [5], and copper [6]. The major difficulty in the separation of calcite from valuable minerals is its fine embedded size and calcite slime. Additionally, the similar physical and chemical properties of the surface of carbonate minerals are another factor that makes separation difficult [7,8,9,10,11]. The shortage of minerals such as smithsonite, fluorite, and scheelite leads to higher and higher requirements for flotation processes and reagents, making the flotation separation of calcite and valuable minerals more difficult. In particular, the difficulty of the effective separation of calcite and valuable minerals is more serious due to calcite slime [12,13]. Jin [13] found that calcite slime covers the surface of smithsonite at a content of 15% through an artificially mixed ore (smithsonite and −10 μm calcite) test, which caused the floatability of smithsonite to change. Zhu et al. [14] also pointed out that calcite slime adsorbed on the surface of smithsonite reduces the interaction of the collectors with the active sites on the mineral surface. Additionally, the poor separation of copper ore in Xiaojiagou is the result of the adsorption or covering of calcite slime on the surface of copper minerals, which affects the action of the collector on the surface of copper minerals [6,15]. Therefore, the actual production process of copper recovery in the Xiaosigou copper is only 50–55%. Eliminating the effects of slime is a hot topic of research at present, and desliming is a direct and effective method [16,17]. There are many studies about calcite slime removal which have achieved good separation.

However, the desliming efficiency is closely related to the type and dosage of metal ions in the solution. It has been found that water quality is the main factor affecting the efficiency of mineral selective desliming [18]. A sufficient amount of Ca^2+^ and Mg^2+^ can cause non-selective flocculation, which makes it difficult to remove the slime effectively [19,20]. When the concentration of Ca^2+^ and Mg^2+^ is greater than 1 × 10^−4^ mol/L under strongly alkaline conditions, it has a more obvious effect on the flocculation and desliming of quartz [21]. The same results were found in the experimental study of the selective flocculation–desliming of smithsonite and limonite (−38 μm) [22]. This indicated that Ca^2+^ and Mg^2+^ dosages affect the selective flocculation and desliming of minerals. Nowadays, many scholars are more concerned with the study of the effect of metal ions on the floatability of slimes (gangue minerals) [23,24] or coal slimes [25,26], or the dispersion behavior of fine silicate minerals [27,28]. However, the effect of metal ions on the dispersion behavior of calcium gangue minerals has rarely been studied. In particular, little research has been reported on the dispersion behavior of −10 μm calcite. Therefore, it is important to identify the mechanism of metal ions on the dispersion behavior of fine calcite to guide the regulation of subsequent desliming.

In this study, calcite, a calcium gangue mineral, was chosen to investigate the effect on the dispersion behavior of micro-fine calcite with different metal ions. It is important to identify the changing pattern of metal ions in fine calcite dispersion behavior, which can be regulated before desliming or flotation dispersion in order to optimize the maximum removal efficiency of calcium gangue minerals and reduce the loss of valuable fine minerals.

## 2. Results and Discussion

### 2.1. Dispersion Behavior Analysis

**Figure 1:**
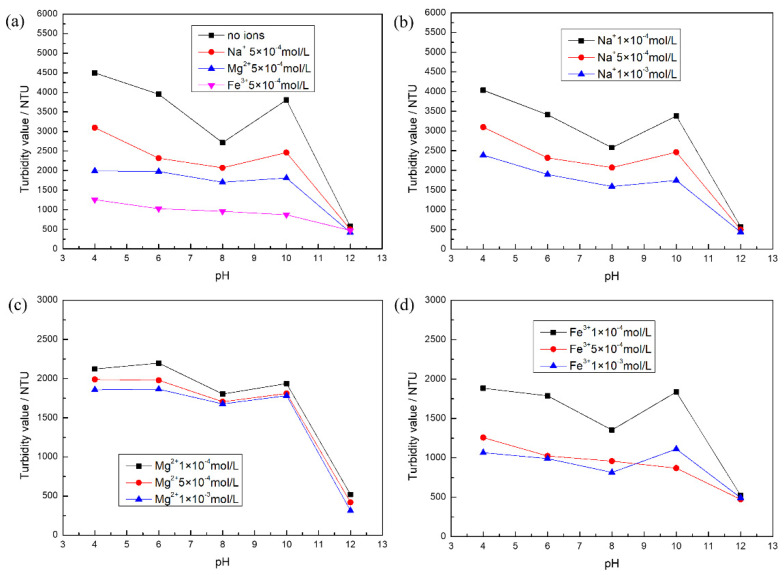
The turbidity test results of calcite: (**a**) different kinds of metal ions; (**b**) different concentrations of Na^+^; (**c**) different concentrations of Mg^2+^; and (**d**) different concentrations of Fe^3+^.

Figure 1a shows that the turbidity value of calcite decreased and then increased in the range of pH = 4–10 in the absence of metal ions, with a minimum value at around pH = 8. However, the turbidity value suddenly became lower with increasing pH. It is possible that the hydrolysis of the trace Ca^2+^ dissolved in calcite produces hydroxide species or metal hydroxide surface precipitation in the interfacial region that reduce the interparticle repulsion by “bridging action” [29]. At pH = 12, calcite particles formed flocs, which accelerated the settling rate, and the lowest turbidity value was measured. However, the dispersion behavior of calcite was different with the addition of different metal ions at a concentration of 5 × 10^−4^ mol/L. The experimental results show that the degree of influence of metal ions on calcite dispersion behavior was Fe^3+^ > Mg^2+^ > Na^+^. The main factor may be that Na^+^ plays the role of a compression electric double layer [27], which changes the electrostatic repulsion between particles. In contrast, Mg^2+^ and Fe^3+^ adsorbed on the mineral surface formed hydroxide species [30,31], which reduced the interparticle interaction potential energy. In particular, the calcite dispersion behavior was completely devastative with 5 × 10^−4^ mol/L Fe^3+^. Therefore, this requires attention to the influence of high-valence metal ions on the dispersion behavior of calcite. Figure 1b shows the consistent trend of the change in the dispersion behavior of calcite for different concentrations of Na^+^ at pH = 4–12. The turbidity values decreased slightly with increasing metal ion dosage, and the Na^+^ dosage had an effect on the dispersion behavior of calcite.

Figure 1c shows the consistent trend of the change in calcite dispersion behavior with different concentrations of Mg^2+^ at pH = 4–12: the measured turbidity values decreased, and the particles formed flocs. Calcite aggregation was more obvious with the increase from Mg^2+^ 5 × 10^−4^ mol/L. When the concentration of magnesium ion is 1 × 10^−3^ mol/L, the measured change in calcite turbidity value was small. This may be due to the small amount of added magnesium ions resulting in a small change in the measured turbidity values of calcite. However, the effects of different concentrations of Fe^3+^ on the dispersion behavior of calcite were different in Figure 1d. At 1 × 10^−4^ mol/L Fe^3+^, the change in calcite dispersion behavior was consistent with the trend of calcite dispersion behavior in the absence of ions, except that the turbidity value decreased. However, the dispersion behavior of calcite was devastated at 5 × 10^−4^ mol/L Fe^3+^. This indicates that the concentration may exceed or be at the critical concentration for the complete destruction of calcite dispersion behavior.

### 2.2. Zeta Analysis

**Figure 2:**
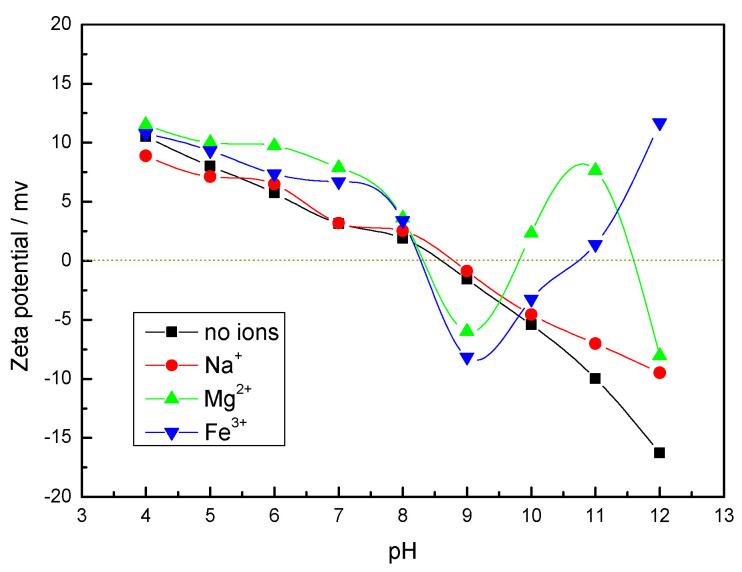
Zeta potential of calcite particles in the presence of metal ions (5 × 10^−4^ mol/L).

The isoelectric point of calcite measured under deionized distilled water in Figure 2 was about pH = 8.5, and the isoelectric point was consistent with the results of the relevant literature [13]. It shows calcite coagulation at around pH = 8. The surface charge of calcite was positive at pH < 8.5. At pH > 8.5, the surface charge of calcite was negative, and the surface electronegativity became more and more negative as the pH increased. This indicates that inter-particle electrostatic force was not a major factor in calcite coagulation under strongly alkaline conditions.

The calcite surface zeta potential changed differently in the presence of different metal ions. There was no change in mineral surface potential positivity and negativity in the presence of Na^+^, but the surface potential shifted positively, and the absolute value of potential become smaller, which was due to the Na^+^ acting as a compressed double layer [27]. However, the mineral surface zeta potential changed considerably in the presence of Mg^2+^ and Fe^3+^, which appeared in multiple isoelectric points. It can be seen in Figure 2 that at pH = 6–8, there is a bulge in the surface potential of calcite under the action of Mg^2+^, which may be the result of the increased action of Mg(OH)^+^. The increasing Mg(OH)_2_ content with increasing pH leads to a positive and negative change in calcite surface potential [32,33]. When the third isoelectric point was reached, the zeta potential of the mineral surface gradually decreased, which may be due to the desorption of metal ions adsorbed on the mineral surface. There is a bump in calcite surface potential at pH = 5–7 which may be due to the action of Fe(OH)_2_^+^ and Fe(OH)^2+^ on iron species. As the pH increases, the Fe(OH)_4_^−^ content increases and the calcite surface potential value decreases. Subsequently, the calcite surface potential value increased, which may be related to the change in the aggregation–dispersion behavior of calcite floc particles [34].

### 2.3. The Solution Chemistry Analysis

In the saturated state, the following equilibrium relationship exists for the dissolution of calcite [33].

(1) $${\mathrm{CaCO}}_{3}\left(s\right){=\mathrm{CaCO}}_{3}\left(\mathrm{aq}\right){\;K}_{1}{=10}^{-5.09}$$

(2) $${\mathrm{CaCO}}_{3}\left(\mathrm{aq}\right){=\mathrm{Ca}}^{2+}{+\mathrm{CO}}_{3}^{2-\;}{\;K}_{2}{=10}^{-3.35}$$

(3) $${\mathrm{CO}}_{3}^{2-}{+H}_{2}{O=\mathrm{HCO}}_{3}^{-}{+\mathrm{OH}}^{-}{\;K}_{3}{=10}^{-3.67}$$

(4) $$H_{2}{O+\mathrm{HCO}}_{3\;}^{-}{=H}_{2}{\mathrm{CO}}_{3}\left(\mathrm{aq}\right){+\mathrm{OH}}^{-}{\;K}_{4}{=10}^{-7.65}$$

(5) $$H_{2}{\mathrm{CO}}_{3}\left(\mathrm{aq}\right){=\mathrm{CO}}_{2}{+H}_{2}{O\;K}_{5}{=10}^{1.47}$$

(6) $${\mathrm{Ca}}^{2+}{+\mathrm{HCO}}_{3}^{-}{=\mathrm{CaHCO}}_{3}^{+}\left(\mathrm{aq}\right){\;K}_{6}{=10}^{0.82}$$

(7) $${\mathrm{CaHCO}}_{3}^{+}{=H}^{+}{+\mathrm{CaCO}}_{3}\left(s\right){\;K}_{7}{=10}^{-7.90}$$

(8) $${\mathrm{Ca}}^{2+}{+\mathrm{OH}}^{-}=\mathrm{Ca}{\left(\mathrm{OH}\right)}^{+}{\;K}_{8}{=10}^{1.4}$$

(9) $$\mathrm{Ca}{\left(\mathrm{OH}\right)}^{+}{+\mathrm{OH}}^{-}=\mathrm{Ca}{\left(\mathrm{OH}\right)}_{2}\left(\mathrm{aq}\right){\;K}_{9}{=10}^{1.37}$$

(10) $$\mathrm{Ca}{\left(\mathrm{OH}\right)}_{2}\left(\mathrm{aq}\right)=\mathrm{Ca}{\left(\mathrm{OH}\right)}_{2}\;\left(s\right){\;K}_{10}{=10}^{2.45}\;$$

In the atmosphere, $P_{{\mathrm{CO}}_{2}}{=10}^{-3.5}\mathrm{atm}$, then $\left[H_{2}{\mathrm{CO}}_{3}\right]{=P}_{{\mathrm{CO}}_{2}}/K_{0\;}{=10}^{-4.97}$, $\log\left(H_{2}{\mathrm{CO}}_{3}\right)=-4.97$, and the relationship between the concentration of each component and pH can be obtained.

**Figure 3:**
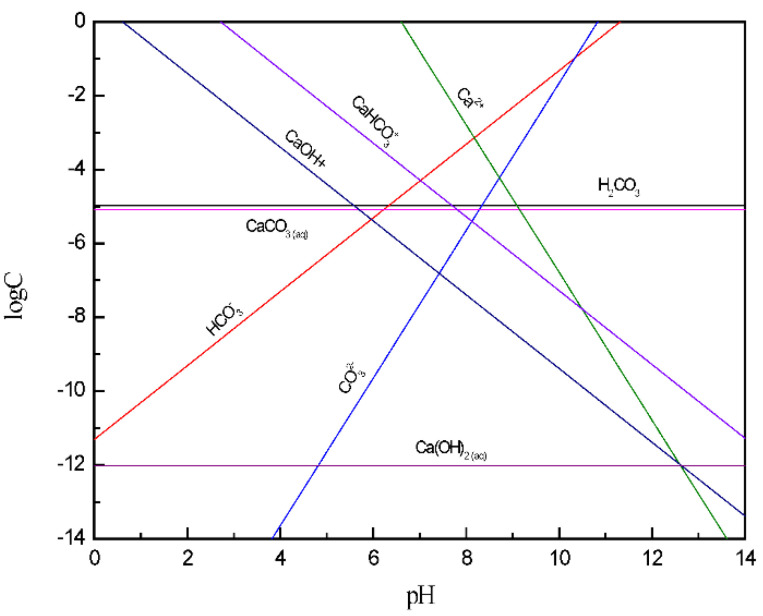
The log C-pH of dissolved components of calcite in solution.

Figure 3 shows that the main components of calcite dissolved ions at pH < 7 are CaHCO_3_^+^, Ca^2+^, and CaOH^+^, which were calcite positioning ions. At this time, the calcite surface potential was positive, the inter-particle force was mainly electrostatic repulsion, and the calcite dispersion behavior was better. At pH > 9, the main components of calcite dissolved ions were CO_3_^2−^ and HCO_3_^−^. At this time, the calcite surface potential was negative and the calcite dispersion behavior was better due to electrostatic repulsion. It is known from the solution chemistry of 5 × 10^−4^ mol/L Ca^2+^ that calcium hydroxide colloids were produced in the solution at pH = 13.04. While [32] KSP’ (calcium hydroxide colloid production on the mineral surface) < KSP (calcium hydroxide colloid formation in solution), so the required pH value was less than 13.04. This indicates that the generated calcium hydroxide adheres to the mineral surface under strongly alkaline conditions, which makes the particles coagulate due to bridging interactions.

The metal ion hydrolysis equilibrium includes a homogeneous system and a multiphase system [33]. Through the equation of metal ion hydration equilibrium in the homogeneous system, the metal ion hydration equilibrium in the multiphase system (when the metal ion generates precipitation) is expressed as follows:

(11) $$N^{n+}=N^{\prime }/{1+\alpha }_{1}{\mathrm{OH}}^{-}{+\alpha }_{2}{\left({\mathrm{OH}}^{-}\right)}^{2\;}{+\alpha }_{n}{\left({\mathrm{OH}}^{-}\right)}^{n}$$

(12) $$\;N{\left(\mathrm{OH}\right)}_{m}^{n-m}{=\alpha }_{m}{\times \;N}^{n+}\times \;{\left({\mathrm{OH}}^{-}\right)}^{m}$$

The calculation formula is obtained by deforming:

(13) $${\;N}^{n+}{=K}_{s0}/{\left({\mathrm{OH}}^{-}\right)}^{n}$$

(14) $$\;N{\left(\mathrm{OH}\right)}_{m}^{n-m\;}{=K}_{\mathrm{sm}}/{\left({\mathrm{OH}}^{-}\right)}^{n-m}$$

where N is a metal element; α_1_, α_2_, … and α_n_ are the accumulation stability constant; and K_s0_, K_s1_ … and K_sm_ are the solubility product. N’ is the sum of the concentration of each component of the metal ion in the solution.

Table 1 shows the stability constants of the hydroxide species of Mg^2+^ and Fe^3+^. Using the data in the table and the above equation, a plot between the concentration of each component of metal ions in solution and log C-pH can be obtained.

**Figure 4:**
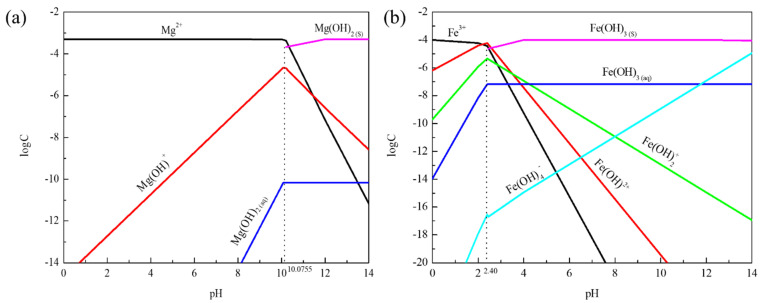
The log-C-pH of hydrolytic components of metal ions in solution: (**a**) Mg^2+^ (5 × 10^−4^ mol/L); (**b**) Fe^3+^ (1 × 10^−4^ mol/L).

From Figure 4a it can be seen that the main component of the solution at pH = 4–10 was Mg^2^. The Mg(OH)^+^ content increased and then decreased with the increase in pH value, which reached a high value of about pH = 10. Subsequently, the Mg(OH)_2_ component was produced at pH = 10.07, while Mg^2+^ and Mg(OH)^+^ components gradually decreased. Mg^2+^ affected calcite dispersion behavior through electrostatic force action under acidic conditions. However, the main reason for the coagulation of mineral particles under alkaline conditions was that Mg(OH)^+^ and Mg(OH)_2_ components promote the coagulation of calcite particles.

When pH < 2, it can be seen from Figure 4b that the main components of the solution were Fe^3+^, Fe(OH)^2+^, and Fe(OH)_2_^+^. The Fe(OH)_3_ component was produced at pH = 2.40. Fe(OH)_3_ and Fe(OH)_4_^−^ gradually increased with the increase in pH value, while the component of Fe^3+^, Fe(OH)^2+^, and Fe(OH)_2_^+^ gradually decreased. Fe(OH)^2+^, Fe(OH)_2_^+^ component was below 10^−9^ orders of magnitude at pH > 6. Fe(OH)_3_ and Fe(OH)_4_^−^ were the main components at pH > 8. The dispersion stability of calcite was devastated, and the particles had severe coagulation whether under acidic or alkaline conditions. It was suggested that Fe(OH)_3_, Fe(OH)^2+^, Fe(OH)_4_^−^, and Fe(OH)_2_^+^ were the main components promoting the coagulation of calcite particles.

### 2.4. SEM, EDS, and XPS Analysis

**Figure 5:**
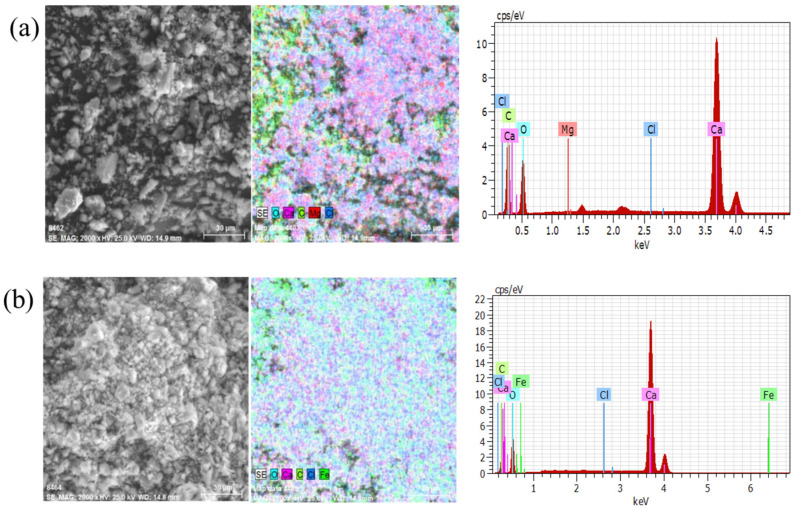
The analysis result of SEM and EDS: (**a**) calcite with Mg^2+^; (**b**) calcite with Fe^3+^.

Many small particles were adhered to form agglomerates in the presence of metal ions from Figure 5a,b. The size of the larger agglomerates was around 30 μm. Platelets were formed between the particles in the presence of Fe^3+^. Meanwhile, the dispersion test results also proved that Mg^2+^ and Fe^3+^ cause calcite serious coagulation.

**Figure 6:**
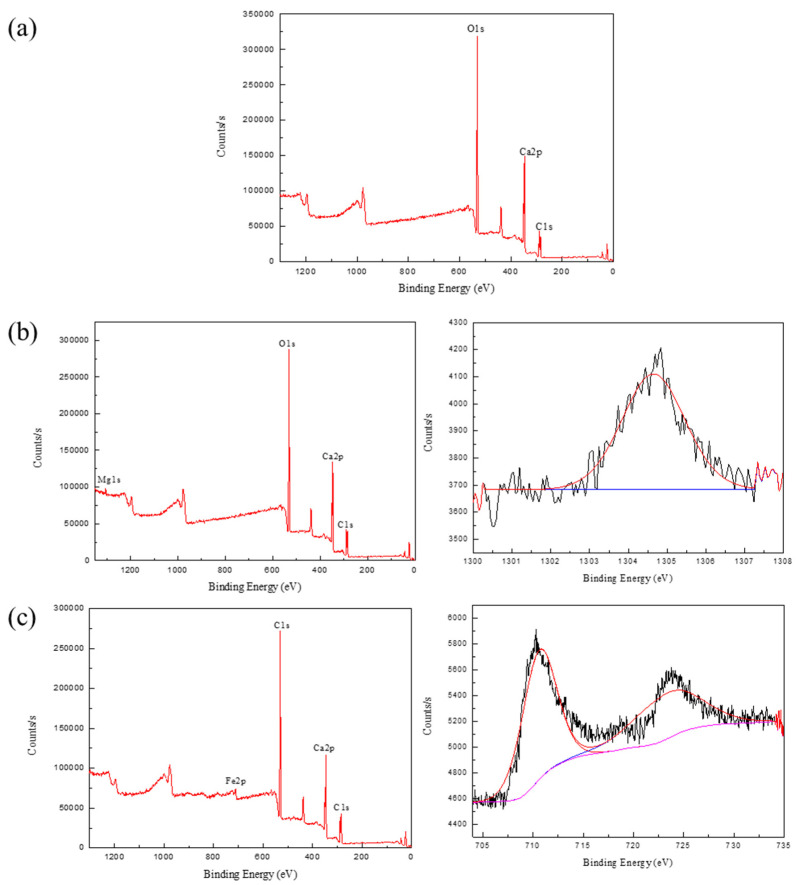
The XPS analysis results of samples: (**a**) calcite; (**b**) calcite with Mg^2+^; (**c**) calcite with Fe^3+^.

As can be seen from Figure 6a, the calcite XPS full spectrum only shows three elemental peaks of calcium, carbon, and oxygen. This indicated that only calcium metal elements were present on the surface of the mineral, without their metal peaks. The results of XPS analysis spectra in the presence of Mg^2+^ and Fe^3+^ from Figure 6b,c had more Mg1s and Fe2p peaks than calcite XPS full spectra results, respectively. The results of the narrow peak mapping of Mg^2+^ and Fe^3+^ also indicated that the calcite mineral surface adsorbed Mg^2+^ and Fe^3+^, and the peak of Fe^3+^ fitting was more obvious than that of Mg^2+^. It is seen from Figure 6b that the Mg1s peak appears in the full spectrum after the action of magnesium ions, and no Mg2p spectral peak appears, which may be the result of the determination of the XPS sample for the turbidity test at pH = 10. It is also possible that the concentration used for the test sample was lower and ended up adsorbed on the mineral surface in smaller amounts. It was also verified that the turbidity value did not change much with a lower concentration of magnesium ions in the turbidity test. It can be found in Figure 6c that the fitted spectra after iron ions’ action at 711.35 eV, 725.07 eV corresponding to Fe2p_3/2_ [35], while the Fe2p_3/2_ binding energy of FeCl_3_ is 711.2 eV, which indicating that Fe(OH)_3_ is generated on the calcite surface [36].

## 3. Experimental

### 3.1. Samples and Reagents

The calcite sample is from the gangue of a lead-zinc deposit in Guangxi Province, China. The D_50_ and D_90_ of the calcite particle size were 5.45 μm, and 12.92 μm, respectively, which were obtained by the S3500 laser particle size analyzer (Microtrac, Montgomeryville, USA). Sodium chloride (NaCl), magnesium chloride (MgCl_2_), and ferric chloride (FeCl_3_) were dissolved to prepare solutions of Na^+^, Ca^2+^, and Fe^3+^ at predetermined concentrations. The reagents were all analytical grade in this study. Deionized distilled water was used in all experiments in order to eliminate the effect of ions in water on calcite dispersion behavior.

### 3.2. XRD and XRF Analysis

**Figure 7:**
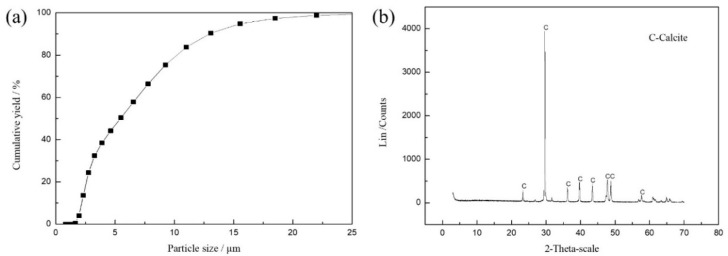
Size distribution (**a**) and XRD analysis spectra (**b**) of calcite.

XRD and XRF were tested by D8 Advance (Bruker, Ettlingen, Germany) and S8 Tiger (Bruker, Ettlingen, Germany) instruments, respectively. The high purity of calcite can be seen in Figure 7b. The purity of calcite is about 99% from Table 2, which meets the test requirements.

### 3.3. Dispersion Experiment Analysis

In a 100 mL beaker a 1 g sample was added to 40 mL deionized water, and they were stirred for 5 min with a magnetic stirrer until fully dispersed. The upper layer (25 mL) of the settling cylinder was extracted for turbidity determination by scattered light turbidimeter (2100AN, HACH, Loveland, CO, USA).

### 3.4. Zeta Analysis

Zeta potential analysis of the sample was performed using the Zeta PALS system (Brookhaven, GA, USA). The 20 mg sample (−5 μm) was taken, the deionized distilled water (50 mL) was added, the pH of the solution was adjusted, and then the metal ions were added. The solution was stirred for 10 min with a magnetic stirrer and left to stand for 12 h. The upper layer of the clear solution was collected in order to determine the zeta potential.

### 3.5. SEM and EDS Analysis

The sample from the dispersion test was vacuum dried and analyzed by SEM and EDS using Quanta 250 (FEI, Hillsboro, OR, USA). The surface of the test sample was gold plated, and mineral surface elemental analysis was performed in face analysis mode.

### 3.6. XPS Analysis

The X-ray photoelectron spectrometer used for the tests was an ESCALAB 250Xi from Thermo Fisher Scientific. The measured mass of the sample (0.5 g) was collected, the deionized distilled water (50 mL) was added, and the pH of the solution was adjusted, and then metal ions were added; the solution was stirred for 10 min with a magnetic stirrer and left to stand for 10 min, then the sample was filtered and vacuum-dried to be XPS measured.

## 4. Conclusions

Under strongly alkaline conditions, the dispersion behavior of calcite in the absence of metal ions was poor, because calcite dissolves. The degree of influence of different metal ions on calcite dispersion behavior was Fe^3+^ > Mg^2+^ > Na^+^. The test of the dosage of the metal ions showed that the dispersion behavior of calcite was poor with increased metal ions dosage. The change in the dispersion behavior of calcite increased with sodium ion dosage. However, the dosage of iron and magnesium ions had a greater impact. In particular, the dispersion behavior of calcite was devastated when the iron ion concentration reached 5 × 10^−4^ mol/L at pH = 4–12. The zeta potential results showed that the calcite surface potential was only positively shifted after the action of sodium ions. Meanwhile, multiple potential isoelectric points appear on the calcite surface after the action of magnesium and iron ions. The solution chemistry analysis showed that these differences in potential values were related to metal hydroxide species or metal hydroxide surface precipitation. The XPS analysis results showed that chemisorption of iron species occurred, and Fe(OH)_3_ was generated on the calcite surface. These changed the calcite dispersion behavior.

## Figures and Tables

**Table 1:** The hydrolytic stability constants of Mg^2+^, Fe^3+^.

Metal Ions	α_1_	α_2_	α_3_	α_4_	K_S0_	K_S1_	K_S2_	K_S3_
Mg^2+^	2.58	1.0	/	/	11.17	8.59	/	/
Fe^3+^	11.81	22.3	32.05	34.3	39.24	27.43	16.94	7.19

**Table 2:** XRF analysis spectra of calcite.

Element	CaCO_3_	MgO	SiO_2_	Al_2_O_3_	Fe_2_O_3_	Other
wt. (%)	99.00	0.69	0.16	0.04	0.02	0.09

## Data Availability

The data presented in this syudy are available in article.

## Sample Availability

Samples of the compounds are available from the authors.
